# Understanding Diabetes Distress Across Adulthood in Type 1 Diabetes: A User‐Centered Design Study

**DOI:** 10.1002/edm2.70331

**Published:** 2026-09-08

**Authors:** Rachel Rayden, Gladys Crespo‐Ramos, Molly Finnan, Claire Hoogendoorn, Todd Farchione, Jeffrey S. Gonzalez

**Affiliations:** ^1^ Albert Einstein College of Medicine New York New York USA; ^2^ Department of Medicine (Endocrinology) Albert Einstein College of Medicine New York New York USA; ^3^ Fleischer Institute of Diabetes and Metabolism Montefiore Medical Center‐Albert Einstein College of Medicine New York New York USA; ^4^ Ferkauf Graduate School of Psychology Yeshiva University New York New York USA; ^5^ Department of Psychological and Brain Sciences Boston University Boston Massachusetts USA; ^6^ Department of Epidemiology and Population Health (Health Behavior Research and Implementation Science) Albert Einstein College of Medicine New York New York USA

**Keywords:** diabetes distress, diabetes technology, type 1 diabetes, young adult

## Abstract

**Aims:**

Diabetes distress is common among adults with Type 1 diabetes (T1D) and is associated with poorer psychological and medical outcomes. While diabetes distress occurs across the lifespan, less is known about how its sources differ by age and how patient input can inform intervention development.

**Methods:**

Using a user‐centred design (UCD) approach, we conducted six virtual group sessions with adults with T1D to inform the development of a cognitive‐behavioural intervention targeting diabetes distress. Participants included young adults aged 18–34 recruited from an academic medical centre and adults aged 35–64 recruited from a national patient advisory council. Sessions were audio‐recorded, transcribed and analysed using a modified inductive qualitative approach.

**Results:**

Nineteen adults with T1D participated. Across age groups, participants identified shared domains of diabetes distress, including competing daily priorities, financial and insurance‐related barriers, interpersonal relationships and healthcare system challenges. However, important age‐related differences emerged. Young adults emphasized immediate disruptions to daily life, identity, competing priorities and technology‐related burden, whereas adults aged 35–64 highlighted cumulative fatigue, fear of complications, caregiving responsibilities and long‐term concerns related to healthcare access and financial sustainability.

**Conclusions:**

Diabetes distress is a pervasive but age‐contextualized experience among adults with T1D. Incorporating stakeholder perspectives revealed both shared and age‐specific drivers of distress, supporting the need for life stage‐responsive psychosocial interventions to address diabetes distress in adults with T1D.

## Graphical abstract

User‐centered design workshops showed that diabetes distress is shaped by life stage. Young adults emphasized competing priorities, technology and identity, while adults aged 35–64 emphasized cumulative burden, caregiving and future planning. These findings informed refinement of a life stage‐tailored psychosocial intervention.

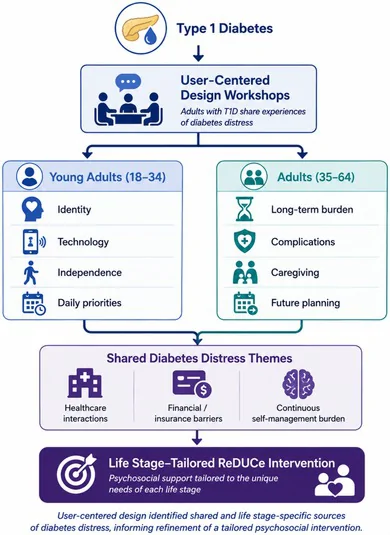

## 1 Introduction

### 1.1 Background and Significance

Type 1 diabetes affects approximately 2.1 million adults in the United States and requires continuous, intensive self‐management to maintain glycemic control and prevent complications [1]. Diabetes‐related distress, defined as the emotional and cognitive burden associated with the ongoing demands of diabetes care, is common among adults with T1D and has been associated with reduced self‐management behaviours, poorer glycemic outcomes and lower quality of life [2]. Unlike clinical depression or anxiety, diabetes distress reflects a condition‐specific response to the relentless and complex nature of living with T1D [3, 4].

Although the prevalence and clinical relevance of diabetes distress are well established [5, 6] and modern measurement approaches have distinguished between the core emotional experience of diabetes distress and the sources that contribute to distress [4, 7, 8], how diabetes distress is experienced across different stages of adulthood in T1D remains relatively unexamined. Given the lifelong nature of T1D, understanding how diabetes distress manifests and evolves across the adult lifespan is essential for developing effective, responsive interventions.

### 1.2 Gap in Knowledge Across the Adult Lifespan

In this study, young adulthood was defined as ages 18–34 to capture a broader developmental period characterized by increasing autonomy, transitions in education and employment, unstable routines and evolving social roles [9, 10, 11]. In contrast, adults in mid‐ to later adulthood (ages 35–64) often face cumulative disease burden, co‐existing medical conditions, caregiving responsibilities and long‐term concerns and financial sustainability [7, 12, 13].

Despite these distinct psychosocial contexts, relatively few studies have directly compared lived experiences of diabetes distress across age groups. Prior research has examined how the challenges of living with Type 1 diabetes evolve across the life course, highlighting changes in self‐management demands, social roles and health priorities from emerging adulthood through later adulthood [11, 14, 15]. However, this literature has focused primarily on self‐management behaviours and clinical outcomes rather than the lived experience of diabetes distress. As a result, psychosocial interventions for diabetes distress are often developed without explicit consideration of life stage‐specific stressors, potentially limiting their relevance and effectiveness [4, 7, 16, 17].

### 1.3 Rationale for the User‐Centered Design Approach

Traditional behavioural interventions for diabetes distress are often developed using researcher‐driven frameworks [16, 18] with limited direct input from individuals living with Type 1 diabetes. User‐centred design (UCD) offers a complementary approach by actively engaging patients as stakeholders in intervention development [19, 20]. Through structured stakeholder engagement methods, UCD can identify contextually grounded sources of distress, cognitive‐behavioural drivers and practical barriers that may not be captured through quantitative measures alone.

Incorporating patient perspectives is particularly important when designing cognitive‐behavioural interventions, as the relevance of cognitive targets, behavioural strategies and intervention examples may vary substantially by age, life context and duration of disease.

To address these gaps, the present qualitative study explored how diabetes distress is experienced across stages of adulthood among individuals with Type 1 diabetes. The workshops were conducted as part of a broader program of research aimed at developing and refining psychosocial interventions to reduce diabetes distress. Insights from these discussions were used to inform intervention development and better understand life stage‐specific challenges related to diabetes distress.

### 1.4 Study Objective

We present a qualitative study using a user‐centered design approach to explore diabetes distress among young adults (ages 18–34) and adults (ages 35–64) with T1D. The age groups were selected to compare diabetes distress across two distinct stages of adulthood characterized by differing developmental, psychosocial and self‐management contexts. Older adults (≥ 65 years), who experience unique clinical and psychosocial challenges, were beyond the scope of the current study and represent an important priority for future research. The objectives were to (1) identify shared and age‐specific sources of diabetes distress and (2) generate stakeholder‐informed insights to guide the refinement and life stage‐appropriate adaptation of cognitive‐behavioural intervention targeting diabetes distress. By comparing themes across age groups, this study contributes to a more developmentally informed understanding of T1D‐related distress and supports the design of tailored behavioural health solutions.

## 2 Subjects, Materials and Methods

### 2.1 Study Design and Setting

We conducted a qualitative descriptive study using a user‐centred design (UCD) approach to inform the development of life stage‐responsive cognitive‐behavioural interventions targeting diabetes distress in adults with Type 1 diabetes. These workshops were conducted during the intervention development phase of two ongoing randomized controlled trials evaluating psychosocial interventions for diabetes distress among adults with Type 1 diabetes, the Tele‐DD and UP‐T1D studies. Methods and reporting followed the Consolidated Criteria for Reporting Qualitative Research (COREQ) guidelines. This work is supported by Breakthrough T1D (4‐SRA‐2021‐1071‐M‐B) and (4‐SRA‐2022‐1187‐M‐B).

The study was approved by the Institutional Review Board of the Albert Einstein College of Medicine (Protocol #2021‐13464). Sample size was guided by the concept of information power [21], which suggests that fewer participants are needed when the study aim is narrow, the sample is information‐rich and the quality of dialogue is expected to be high. We planned four young adult workshops and two adult workshops to capture perspectives within each predefined age group while allowing for recurring themes to emerge across multiple discussions. Thematic sufficiency was monitored throughout data collection to determine whether additional sessions were needed.

### 2.2 Participants and Eligibility Criteria

Young adults were eligible if they (1) had a clinical diagnosis of Type 1 diabetes, (2) were aged 18–34 years, (3) were English‐speaking and (4) were able to participate in a 90 min virtual group session. Exclusion criteria included acute medical instability requiring hospitalization within the prior 30 days or cognitive impairment precluding informed consent.

Adults aged 35–64 years met identical inclusion and exclusion criteria except for the age range. Age group cut‐offs were selected to reflect developmental and clinical differences across early and established adulthood, including transitions in autonomy, disease duration and cumulative self‐management burden. The upper limit of 34 years was chosen to capture the extended transition to stable adult roles that has been described in prior diabetes and developmental literature while maintaining a meaningful comparison with adults aged 35–64 years.

### 2.3 Recruitment and Consent

Young adults were recruited from the adult and paediatric diabetes clinics at the Children's Hospital at Montefiore and Fleischer Institute for Diabetes and Metabolism at Montefiore Medical Center in the Bronx, NY. Adults aged 35–64 were recruited from Breakthrough T1D's national Patient Advisory Council. Potential participants were approached via clinician referral or registry‐based email and phone outreach. Verbal informed consent was obtained and documented prior to participation. Participants received a $100 gift card for participation.

### 2.4 User‐Centered Design Sessions

Six virtual UCD sessions were conducted: four with young adults and two with adults aged 35–64. Each session lasted approximately 90 min and was facilitated by a clinical psychologist, with support from a research coordinator. Sessions followed a semi‐structured facilitation guide developed by the study team.

Sessions included two primary components. First, participants were prompted to describe how diabetes distress affected their daily lives, routines, relationships and interactions with the healthcare system. Second, participants were introduced to core cognitive‐behavioural concepts underlying the proposed intervention and invited to provide feedback on the relevance, clarity and feasibility of candidate components. Sessions incorporated brief individual reflection followed by group discussion to allow participants to generate personal insights prior to shared dialogue. Although workshops were intended to include several participants, attendance varied because of scheduling constraints, resulting in smaller discussion groups rather than originally planned. All workshops followed the same discussion guide and facilitation procedures regardless of group size.

Findings from these workshops were used to refine the content and structure of the Reduce intervention, including the selection of session examples, discussion prompts and cognitive‐behavioural strategies tailored to life stage‐specific sources of diabetes distress. For example, participant feedback informed the inclusion of age‐relevant scenarios addressing technology‐related burden and competing priorities for young adults and cumulative burden, caregiving responsibilities and long‐term planning for adults aged 35–64.

### 2.5 Data Collection and Analysis

All English‐language sessions were audio‐recorded and professionally transcribed. To preserve patient confidentiality, transcripts were de‐identified prior to analysis and demographic characteristics were not linked to individual quotes. Transcripts were reviewed for accuracy and uploaded into NVivo 12 for analysis. Validated diabetes distress questionnaires were not administered, as the primary objective of this user‐centred design study was to elicit participants' lived experiences and inform intervention development through qualitative stakeholder input rather than quantify distress severity. The analytic approach followed a modified inductive coding process.

Two members of the research team (RR and MF) independently reviewed transcripts to identify initial codes and develop a coding framework. All transcripts were coded by one analyst (MF), with a second analyst (RR) independently coding 20% of transcripts to assess consistency. Discrepancies were resolved through discussion and the coding framework was iteratively refined. Codes were grouped into categories and synthesized into broader themes representing domains of diabetes distress. An audit trail was maintained to document analytic decisions and enhance rigour.

### 2.6 Methodological Rigour

Several strategies were used to enhance trustworthiness, including use of a standardized facilitation guide, team‐based coding and consensus procedures and maintenance of an audit trail. Brief end‐of‐session summaries were used to confirm key takeaways with participants. Formal transcript member checking was not conducted to minimize participant burden.

## 3 Results

### 3.1 Participant Characteristics

Nineteen adults with Type 1 diabetes (T1D) participated in six virtual user‐centred design sessions, including 14 young adults aged 18–34 years and 5 adults aged 35–64 years. Participant characteristics are summarized in Table 1. Young adults had a mean age of 23.8 years and 64.3% were female, whereas all participants in the 35–64 age group were female. Participants were recruited from both an academic medical centre and a national patient advocacy organization, representing individuals actively engaged in diabetes care and self‐management.

**TABLE 1:** Participant characteristics of adults with Type 1 Diabetes in User‐Centered Design sessions.

Characteristics	Young adults (18–34 years) (*n* = 14)	Adults (35–64 years) (*n* = 5)	Total (*N* = 19)
Age, mean (range), years	23.8 (19–31)	45.5 (35–58)	
Female sex, *n* (%)	9 (64.3)	5 (100)	14 (73.7)
Hispanic/Latino, *n* (%)	6 (42.9)	1 (20)	7 (36.8)
Black/African American, *n* (%)	6 (42.9)	0 (0)	6 (31.6)
European American, *n* (%)	2 (14.3)	4 (80)	6 (31.6)
User‐centred design sessions	4	2	6

### 3.2 Overview of Diabetes Distress Across Age Groups

Both young adults and adults aged 35–64 identified competing daily priorities as a central contributor to diabetes distress. Financial and insurance‐related barriers were also prominent across age groups, with participants emphasizing not only the cost of supplies and medications but also the emotional burden associated with navigating insurance requirements, prior authorizations and recurrent administrative hurdles. Although financial and insurance‐related challenges were common across both age groups, participants tended to contextualize these stressors differently according to their stage of adulthood, with younger adults discussing their impact on day‐to‐day independence and older adults emphasizing long‐term affordability and sustainability of care. Interpersonal relationships emerged as another shared domain of distress. Participants described strain within professional, social and family relationships related to the visibility of diabetes, perceived misunderstanding by others and the need to repeatedly explain or justify diabetes‐related needs. Interactions with the healthcare system were also described as emotionally taxing, with participants reporting feelings of judgement, frustration or limited attention to emotional well‐being during clinical encounters. Taken together, participants described diabetes distress as arising not from isolated diabetes tasks but from the cumulative challenge of integrating diabetes management into the competing demands of everyday life, relationships, work and interactions with healthcare systems.

### 3.3 Age‐Specific Experiences of Diabetes Distress

#### 3.3.1 Young Adults (Age 18–34)

Young adults emphasized the immediate and intrusive nature of diabetes distress, particularly how diabetes disrupted daily routines, social interactions and emerging adult identities. Rather than describing diabetes as a series of discrete management tasks, participants portrayed it as a constant background presence that shaped everyday decisions and influenced how they navigated work, school, relationships and social situations. Distress in this group centered on the constant cognitive load of diabetes decision‐making and the challenge of balancing self‐management with competing personal and professional priorities. Participants described silencing alarms, delaying treatment or minimizing visible diabetes care during meetings or social situations to avoid disruption or unwanted attention.

Technology‐related burden was particularly salient among young adults. Participants expressed frustration related to device malfunction, alarm fatigue and the persistent visibility of diabetes technology, which often contributed to feelings of stigma or being ‘different’ from peers. These experiences were frequently described as emotionally exhausting and contributed to disengagement from optimal self‐management behaviours. Themes and exemplar quotes from young adults are presented in Table 2.

**TABLE 2:** Themes and exemplar quotes describing diabetes distress among young adults with Type 1 Diabetes.

Theme	Frequency (*n*)	Exemplar quote
Daily burden	19	‘There's just a responsibility that people don't recognize or realize that we have.’
Mental burden/cognitive load	16	‘We work so hard just to stay alive, but that's not enough.’
Competing priorities	15	‘I just keep silencing my alarm…I'll deal with this when I'm done.’
Technology‐related distress	13	‘Device fatigue—yes. There are days I want to throw this thing.’
Stigma and social visibility	12	‘We're the token diabetes people. When people see my devices, they have all these questions.’
Interpersonal relationships	12	‘Your work relationships are different, your friendships are different because of diabetes.’

*Note:* Frequency reflects number of coded excerpts across sessions, not number of participants.

#### 3.3.2 Adults Aged 35–64

Adults, aged 35–64, described diabetes distress as a cumulative and future‐oriented burden shaped by long‐term disease management, co‐existing medical conditions and concerns about sustaining care over time. Many participants reflected on how their experiences of diabetes distress had evolved over decades, with ongoing self‐management becoming intertwined with changing health concerns, family responsibilities and planning for the future. Participants emphasized the emotional toll of decades of continuous self‐management, often describing persistent mental fatigue and vigilance that felt necessary but rarely affirming.

Fear of complications and the physical ‘wear and tear’ of long‐standing diabetes were prominent sources of distress in this group. Participants also highlighted caregiving responsibilities and long‐term financial planning, including concerns related to retirement and healthcare access. Interactions with healthcare providers were frequently described as judgement‐laden or emotionally uncomfortable, particularly when emotional burden was perceived as secondary to glycaemic metrics. Themes and exemplar quotes from adults aged 35–64 are presented in Table 3.

**TABLE 3:** Themes and exemplar quotes describing diabetes distress among adults with Type 1 diabetes.

Theme	Frequency (*n*)	Exemplar quote
Healthcare team relations	24	‘When you're going to meet with the endo, it's like sitting in the principal's office.’
Financial burden	8	‘I could buy a house with what I've spent to keep myself alive.’
Insurance logistics	8	‘We know I still have diabetes—it's not cured this year.’
Co‐existing conditions and fear of complications	5	‘Living with this constant wear and tear…the fear of complications.’
Mental burden	16	‘The extra 300 decisions we get to make every day because of T1D.’
Interpersonal relationships and caregiving	12	‘Asking for help… Can you get me a juice instead of me having to get the juice?’

*Note:* Frequency reflects number of coded excerpts across sessions, not number of participants.

### 3.4 Conceptualization of Diabetes Distress

Across age groups, participants described diabetes distress as a chronic, disease‐specific emotional and cognitive burden arising from the continuous demands of managing T1D. Distress was clearly distinguished from depression or anxiety and instead framed as an expected response to living with a complex, lifelong condition. Participants emphasized that diabetes distress encompassed daily self‐management tasks, emotional responses to glycaemic variability, healthcare system interactions and structural barriers such as insurance and cost.

As one participant noted, ‘almost everything that a Type 1 goes through has the potential to cause distress,’ including difficulty obtaining supplies, navigating insurance requirements and accessing timely care.

#### 3.4.1 Cross‐Age Synthesis

In summary, while young adults and adults aged 35–64 shared core domains of diabetes distress, the lived experience and framing of distress differed by life stage. Although financial strain, healthcare barriers and competing demands were discussed across both groups, participants differed in how these challenges were experienced and prioritized within their broader life contexts. For young adults, diabetes distress was commonly framed around establishing independence, balancing competing daily responsibilities and navigating social identity. Adults aged 35–64 more often framed distress in relation to sustaining long‐term self‐management, caregiving responsibilities and concerns about future health and financial security. Despite many common stressors across groups, participants consistently interpreted these experiences through the lens of their current stage of life, with developmental roles and responsibilities shaping both the sources and meaning of diabetes distress. Young adults emphasized immediacy, identity disruption and technology‐related burden, whereas adults aged 35–64 highlighted cumulative fatigue, fear of complications, caregiving demands and long‐term healthcare and financial concerns. These findings underscore the importance of considering life stage when designing and implementing psychosocial interventions for adults with T1D.

## 4 Discussion

In this qualitative study using a user‐centred design approach, both young adults and adults aged 35–64 with T1D described diabetes distress as a pervasive, condition‐specific burden embedded in daily life. Consistent with prior literature, participants clearly distinguished diabetes distress from depression and anxiety, framing it instead as an expected response to the relentless cognitive, emotional and logistical demands of managing T1D [7, 22]. Importantly, this study extends prior qualitative work by demonstrating that, while core domains of diabetes distress are shared across adulthood, the meaning, salience and temporal framing of distress differ systematically by life stage.

### 4.1 Shared Drivers of Diabetes Distress

Across age groups, participants identified several shared drivers of diabetes distress, including the daily burden of self‐management, competing priorities, healthcare system interactions and financial and insurance‐related barriers. The prominence of healthcare‐related distress, particularly perceptions of judgement, limited attention to emotional burden and focus on glycaemic metrics, highlights persistent gaps in the integration of psychosocial care into routine diabetes management [4, 16]. These findings align with prior evidence linking negative healthcare interactions to increased diabetes distress and disengagement from care.

Importantly, financial and insurance‐related stressors were described not only as economic concerns, but as ongoing sources of administrative and emotional burden. Participants emphasized the distress associated with navigating insurance coverage, prior authorizations and access to supplies, experiences that compounded emotional fatigue and reinforced feelings of burnout. Together, these findings suggest that diabetes distress is shaped not only by the demands of daily self‐management, but by sustained friction between individual care efforts and healthcare systems that are perceived as inflexible or unsupportive, underscoring the need for interventions that acknowledge and address systemic contributors to diabetes distress.

### 4.2 Life Stage‐Specific Experiences of Diabetes Distress

Despite shared domains of diabetes distress, meaningful differences emerged in how distress was experienced and contextualized across adult life stages. Young adults emphasized immediacy and disruption, describing diabetes as intruding upon daily routines, social interactions and emerging adult identities. These findings suggest that diabetes distress was shaped not only by age but by the developmental context in which participants were managing T1D. Young adults frequently described navigating diabetes alongside major educational, occupational and social transitions, making the visibility and day‐to‐day demands of diabetes particularly disruptive to establishing independence and identity. Competing priorities, technology‐related burden and the visibility of diabetes management were central to distress in this group. These findings are consistent with prior work demonstrating heightened diabetes distress during emerging adulthood, a period marked by instability, autonomy development and role transitions [9, 10].

In contrast, adults aged 35–64 described diabetes distress as cumulative and future‐oriented, shaped by long‐term disease management and evolving life responsibilities. Rather than emphasizing the immediate disruptions of diabetes, participants more often reflected on how decades of disease management intersected with caregiving responsibilities, long‐term health concerns and planning for the future, illustrating how the meaning of diabetes distress evolves across adulthood. Participants highlighted persistent mental fatigue, fear of complications, co‐existing medical conditions, caregiving responsibilities and concerns about sustaining care over time. Distress in this group was often shaped by decades of continuous self‐management and uncertainty related to aging, retirement and healthcare access. Together, these findings suggest that diabetes distress is influenced not only by disease duration, but by shifting developmental contexts and future orientation across the adult life course, extending prior work that has primarily focused on early adulthood [7].

### 4.3 Implications for Intervention Development

These findings have direct implications for the design of psychosocial interventions targeting diabetes distress. While core components of diabetes distress interventions, such as emotional validation, cognitive restructuring and problem‐solving, appear broadly relevant across age groups, our findings suggest that intervention content and emphasis should be adapted to life stage‐specific stressors to optimize engagement and relevance. Insights from these workshops directly informed the refinement of the Reduce intervention, including the development of age‐specific examples, discussion prompts and cognitive‐behavioural strategies tailored to life stage‐specific sources of diabetes distress. For example, participant feedback informed intervention content addressing technology fatigue, competing priorities and identity‐related concerns among young adults, while emphasizing cumulative burden, caregiving responsibilities and long‐term planning for adults aged 35–64.

For young adults, interventions may be strengthened by explicitly addressing competing priorities, technology‐related fatigue, stigma and identity‐related challenges. For adults aged 35–64, greater emphasis on cumulative burden, fear of complications, caregiving demands and long‐term planning may better align with lived experience.

### 4.4 Broader Context Within the Diabetes Distress Literature

The findings of this study align with and extend prior literature conceptualizing diabetes distress as a dynamic, context‐dependent experience rather than a static psychological state. While previous work has demonstrated that diabetes distress fluctuates over time and in response to changing life circumstances, treatment demands and healthcare environments [4, 7] and other studies have shown that diabetes distress remains stable over time when assessed over longer time frames (3–6 months) [23], this study adds nuance by illustrating how the meaning and framing of distress shift across adult life stages. By directly eliciting patient perspectives across adult life stages, this study contributes novel insight into how the sources and framing of distress evolve with age and accumulated disease experience. These findings are consistent with broader conceptual models and syntheses of the diabetes distress literature [24, 25].

Prior qualitative research has also played a central role in shaping how diabetes distress is conceptualized and measured. For example, qualitative interviews with adults with Type 1 diabetes informed the development of the Type 1 Diabetes Distress Scale (T1‐DDS) and subsequent refinements in the Diabetes Distress Assessment System, identifying domains such as regimen burden, interpersonal distress and physician‐related distress as core sources of distress [8, 26]. Similarly, qualitative work in Type 2 diabetes has identified treatment regimen demands, interpersonal dynamics and healthcare interactions as key drivers of distress from the patient perspective [27].

Our findings are consistent with these prior qualitative studies in highlighting the central role of daily management demands and healthcare interactions in shaping diabetes distress. However, the present study extends this literature by examining how the salience and framing of these domains vary across stages of adulthood, emphasizing the importance of life stage‐specific context in understanding diabetes distress.

Participants**'** narratives further reinforce conceptual models that position diabetes distress as relational and systemic, shaped not only by individual coping efforts but also by interactions with healthcare systems, insurers, technology, workplaces and family contexts [3, 28]. The prominence of insurance navigation, administrative burden and perceived judgement from healthcare providers underscores that diabetes distress is often generated or amplified by modifiable system‐level factors rather than personal failure.

### 4.5 Clinical Implications for Screening and Care Delivery

These findings have important implications for routine screening and clinical conversations about diabetes distress. Although validated screening tools such as the Diabetes Distress Scale are widely used, participants' narratives suggest that distress may be underestimated when assessments focus narrowly on emotional symptoms without sufficient attention to contextual stressors such as cost, caregiving responsibilities or technology burden [7, 29]. Incorporating brief, open‐ended, patient‐centered questions into routine encounters by endocrinologists, diabetes educators or interdisciplinary care teams may improve identification of distress that is situational, cumulative or anticipatory in nature.

Additionally, the recurrent theme of perceived judgement during clinical visits underscores the importance of trauma‐informed and non‐punitive approaches to diabetes care. Prior work has shown that provider communication style is strongly associated with diabetes‐related emotional outcomes, adherence and trust [17, 30]. Efforts to reduce diabetes distress may therefore benefit from parallel strategies that support clinician training in empathetic communication and shared decision‐making, alongside patient‐focused interventions.

### 4.6 Relevance to Technology Use and Treatment Burden

In this study, technology‐related distress emerged as a particularly salient source of burden among young adults, occurring alongside the rapid expansion of diabetes technologies, including continuous glucose monitoring and automated insulin delivery systems. While these tools offer substantial clinical benefits, participants described how they may simultaneously introduce emotional and cognitive burden, including alarm fatigue, data overload and the persistent visibility of diabetes in daily life [31, 32]. These findings support calls for more nuanced discussions of ‘treatment burden’ in T1D, emphasizing the emotional trade‐offs associated with advanced therapies [33, 34].

Among adults aged 35–64, treatment burden was more commonly described in relation to long‐term sustainability of care, aging with diabetes and fear of complications, echoing prior literature highlighting the intersection of diabetes distress with aging, multimorbidity and caregiving roles [12, 35]. Addressing diabetes distress in this population may therefore require integrating psychosocial support within broader models of chronic disease self‐management and care for aging adults with complex health needs.

### 4.7 Implications for Future Research

Future research should build on these findings by examining diabetes distress longitudinally across adulthood and by explicitly evaluating life stage as a moderating factor in psychosocial intervention trials. Quantitative studies may benefit from stratifying analyses by age group, disease duration or both, to better capture heterogeneity in distress experiences. Additionally, greater inclusion of racially, ethnically and socioeconomically diverse populations is essential, given well‐documented disparities in diabetes outcomes and access to care [36, 37]. Older adults (≥ 65 years) were not represented in this study and warrant dedicated investigation, as aging with Type 1 diabetes introduces unique clinical, psychosocial and self‐management challenges that may shape the experience of diabetes distress.

More systematic integration of user‐centred design approaches earlier in intervention development may further enhance the acceptability, relevance and sustainability of psychosocial programs targeting diabetes distress. As emphasized by participants in this study, interventions that explicitly acknowledge lived experience and structural barriers may be better positioned to support meaningful engagement across the adult lifespan.

### 4.8 Strengths and Limitations

Strengths of this study include the use of rigorous qualitative methods, direct incorporation of stakeholder perspectives and comparison of diabetes distress across adult life stages. Additional strengths include the application of a user‐centred design approach to inform intervention development. Limitations include a small sample size and smaller workshop sizes than originally intended because of scheduling constraints, which may have limited opportunities for group interaction and discussion. Furthermore, all participants in the 35–64 age group were female, which may limit the transferability of these findings to men with Type 1 diabetes in this age range. Future studies should include more diverse samples to better characterize potential sex‐ and gender‐related differences in the experience of diabetes distress across adulthood. Additionally, participants were not assessed using a validated diabetes distress instrument, limiting our ability to characterize distress severity or compare findings across established clinical thresholds. Participants were also largely recruited from clinical and advocacy settings, which may limit generalizability to individuals who are less engaged with diabetes care. Future research should examine how diabetes distress intersects with gender, race, socioeconomic status and access to care.

## 5 Conclusion

Both young adults and adults with Type 1 diabetes experience diabetes distress, though important differences exist in how distress is experienced and contextualized across the life course. Participants in both age groups described rich lived experiences of diabetes distress and identified specific domains that contributed most to their burden, including competing daily priorities, financial and insurance‐related barriers, interpersonal relationships and provider‐ and system‐level challenges. While these stressors were shared, their salience and framing differed by life stage, reflecting distinct developmental, social and healthcare contexts. These findings underscore the importance of life stage‐responsive psychosocial approaches to diabetes distress, as adapting interventions to individuals' lived experiences across the lifespan may enhance engagement and improve effectiveness among adults with Type 1 diabetes.

## Author Contributions

**Rachel Rayden:** investigation, writing – original draft, methodology, writing – review and editing, data curation. **Gladys Crespo‐Ramos:** investigation, methodology, writing – review and editing. **Molly Finnan:** investigation, methodology, writing – review and editing. **Claire Hoogendoorn:** writing – review and editing. **Todd Farchione:** writing – review and editing. **Jeffrey S. Gonzalez:** writing – review and editing, supervision.

## Funding

This work is supported by Breakthrough T1D (4‐SRA‐2021‐1071‐M‐B) and (4‐SRA‐2022‐1187‐M‐B).

## Conflicts of Interest

The authors declare no conflicts of interest.

## Data Availability

Data sharing not applicable to this article as no datasets were generated or analysed during the current study.
